# Dental Plaque Removal by Ultrasonic Toothbrushes

**DOI:** 10.3390/dj8010028

**Published:** 2020-03-23

**Authors:** Ilya Digel, Inna Kern, Eva Maria Geenen, Nuraly Akimbekov

**Affiliations:** 1 Laboratory for Cell- and Microbiology, FH Aachen University of Applied Sciences, 52428 Juelich, Germany; inna.kern@alumni.fh-aachen.de (I.K.); geenen@fh-aachen.de (E.M.G.); 2 Department of Biotechnology, al-Farabi Kazakh National University, 050040 Almaty, Kazakhstan; akimbekov.nuraly@kaznu.kz

**Keywords:** dental plaque removal, ultrasonic toothbrushing, dental biofilms, powered toothbrushes

## Abstract

With the variety of toothbrushes on the market, the question arises, which toothbrush is best suited to maintain oral health? This thematic review focuses first on plaque formation mechanisms and then on the plaque removal effectiveness of ultrasonic toothbrushes and their potential in preventing oral diseases like periodontitis, gingivitis, and caries. We overviewed the physical effects that occurred during brushing and tried to address the question of whether ultrasonic toothbrushes effectively reduced the microbial burden by increasing the hydrodynamic forces. The results of published studies show that electric toothbrushes, which combine ultrasonic and sonic (or acoustic and mechanic) actions, may have the most promising effect on good oral health. Existing ultrasonic/sonic toothbrush models do not significantly differ regarding the removal of dental biofilm and the reduction of gingival inflammation compared with other electrically powered toothbrushes, whereas the manual toothbrushes show a lower effectiveness.

## 1. Introduction

Dental plaque (also called microbial plaque, oral biofilm, dental biofilm) includes highly organized diverse microbial communities attached to the surface of hard tooth tissues. Oral biofilm’s role in the development of various diseases of the oral cavity and throat, including caries, periodontal diseases, endodontic infections, tonsillitis, alveolitis, among others, determines the clinical and biological relevance of studying this system [1]. Moreover, due to its peculiar “biogeography”, it is very suitable as a model of an integrated biological microecosystem.

Biofilm consortia are characterized by unique architecture and specific features of adhered cells, which are physiologically, metabolically, and morphologically different from their planktonic counterparts [2]. Microbial populations within the biofilm are involved in a chain of physical, metabolic, and molecular interactions that can modulate antibiotic resistance and pathogenicity. Boles et al. suggested that diversity in biofilms provides a form of “biological insurance” thereby allowing microbial cells to resist adverse conditions [3]. The antibiotic resistance of the biofilm is associated with several factors, i.e., the ability of the extracellular matrix to serve as the first line of defense against antibiotic attack and the facilitated gene transfer among microorganisms, among others. [4]. It was reported that microbial cells fixed in the biofilm are 10–1000 times more resistant to antibiotics than the planktonic cells [5].

The biofilm formation is a gradual process consisting of several successive stages: (a) acquired pellicle formation before adhesion of planktonic microflora; (b) primary (early) colonization with subsequent proliferation of adhered microorganisms; (c) secondary colonization/co-aggregation; and (d) biofilm maturation [6].

**Figure 1:**
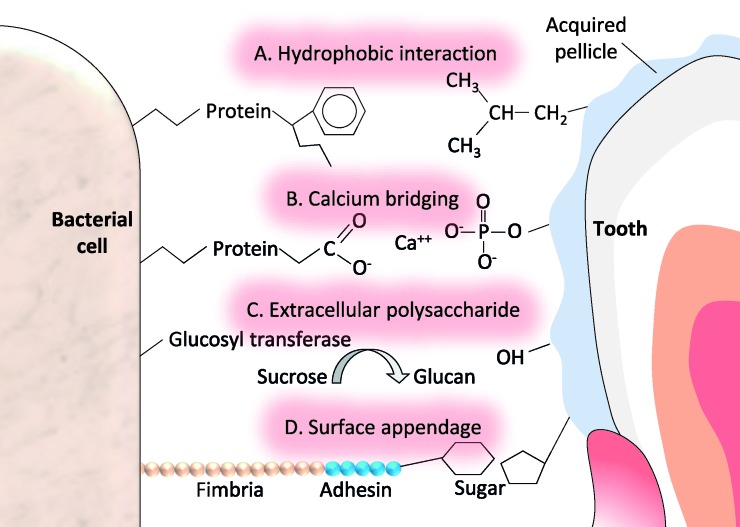
Possible molecular mechanisms underlying bacterial attachment to teeth during dental plaque formation. A. Hydrophobic interaction (between a side chain of a phenylalanine component of a bacterial protein and a side chain of a leucine component of a salivary glycoprotein in the acquired pellicle). B. Calcium bridging (between a negatively charged carboxyl group of a bacterial protein and a positively charged calcium ion, i.e., electrostatic attraction). C. Extracellular polysaccharide (the host’s dietary sucrose is converted by bacterial glucosyltransferase to glucan, which has many functional groups and can interact with amino acid side-chain groups (serine, tyrosine, and threonine). D. Surface appendages (bacterial fimbria extend to permit the terminal adhesin portion to bind to a sugar component of a salivary glycoprotein in the acquired pellicle).

The initial bacterial attachment to the acquired pellicle involves physicochemical and biochemical interactions (e.g., electrostatic forces and hydrophobic bonding) between molecules present at the tooth surface and those present on the cell surface (Figure 1).

The combination of the primary adhesion, co-adhesion, and co-aggregation of bacteria accompanied by their rapid proliferation leads to effective colonization of host tissues and the quick formation of the dental microbial biofilm [7,8]. Biofilm development proceeds under the participation of both adhered and planktonic microorganisms, and its further maturations are dominated by growth and differentiation of the constituting microorganisms [9].

Mature biofilms are represented by a multilayer structure with a heterogeneous population of cells surrounded by an extracellular polymeric substance (EPSs). Channels are penetrating the biofilm matrix, in which nutrients can circulate and metabolic waste products can be excreted. The EPSs consist of exopolysaccharides and proteins, as well as other macromolecules, such as nucleic acids, and lipids. The exopolysaccharide matrix provides versatile protection against toxins, pH and osmotic changes, ultraviolet radiation, and insiccation. Once embedded within a biofilm, bacteria gain resistance to the host immune system [10].

The supragingival environment is colonized with species such as *Staphylococcus intermedius*, *Streptococcus oralis*, *S. mitis*, *S. mutans*, *S. anginosus*, *Selenomonas noxia*, *Veillonella parvula*, *Capnocytophaga gingivalis*, *Eikenella corrodens*, *Neisseria mucosa*, *Fusobacterium nucleatum*, *Treponema sokranskii*, *Prevotella melaninogenica*, *Propionibacterium acnes*, and *Leptotrichia buccalis*; while the subgingival biofilm is composed predominantly of *F. nucleatum*, *Campylobacter rectus*, *V. dispar*, *Porphyromonas gingivalis*, *P. intermedia*, *Tannerella forsythia*, *T. denticola*, *Actinomyces oris*, *S. anginosus*, and *S. oralis* [11]. These microbial communities can produce high concentrations of metabolites, such as acids, ammonia, carbon dioxide, as well as various oxidants such as hydrogen peroxide, which can negatively affect host immunity in the oral cavity.

On the one hand, the biofilm can be considered as a commensal part of the natural immune system; while on the other hand, uncontrolled activities of pathogenic bacteria can cause health problems like gingival inflammation, characterized by redness of the gingiva, swelling, and bleeding [12]. The infiltration of the periodontal membrane by progressing gingival inflammation can cause periodontitis—a multifactorial disease having high epidemiological significance. Therefore, the removal of supra- and sub-gingival biofilm is mostly beneficial for periodontal health in the long term and is directly related to effective toothbrushing [13]. Currently, there are no means to ensure the complete and final removal of dental plaque/biofilms from the oral cavity. Nevertheless, its pathogenicity can be significantly reduced by violating the integrity of the structure and restoring normal microflora using conventional dentifrices, cleansing agents, and different toothbrushes. Especially, ultrasonic toothbrushes appear to remove dental plaque effectively and can considerably improve oral health.

In general, the efficiency of toothbrushing is dependent on the type of toothbrush, wearing of toothbrush [14], method of brushing [15], time of brushing, and on the use of mouthwashes [16] and/or dental floss [17]. In self-performed oral hygiene, the main concern is the removal of biofilm in interdental areas such as supra- and sub-gingival biofilm. While different methods exist for the prevention of plaque formation, the use of toothbrushes has proven to be the most efficient way [18].

In the early 1960s, powered toothbrushes were first introduced for commercial use and have represented an alternative to manual toothbrushing [12]. Since then, electrical-powered toothbrushes have become increasingly popular which raises the question of which type of toothbrush performs better.

The bristle motion of the toothbrush at different frequencies, apart from the direct scratching effect, generates a turbulent fluid flow that directly leads to hydrodynamic effects such as wall shear forces that act parallel to the tooth surface [19]. Electrically powered toothbrushes may help to perform adequate oral hygiene due to the application of higher frequencies as compared to manual brushes [13]. Ultrasonic toothbrushes mainly differ from conventional electrical toothbrushes in their even higher operating frequency (>20 kHz) [20]. The used frequency range is not audible for the human ear and may be beneficial since hydrodynamic forces (such as the flow rate of the dental fluid and the formation of bubbles) are significantly increased [13]. Still, the exact relation between the energy transfer from the brush to the biofilm and the contribution of acoustic waves to biofilm removal remain unclear [21].

Now we will try to address the issue regarding the effectiveness of ultrasonic toothbrushes in terms of their technical characteristics and in terms of the various hydrodynamic forces acting on the biofilm. Further, the performance of manual, sonic, and ultrasonic toothbrushes will be compared briefly regarding the reduction of plaque, gingival inflammation, and the number of bacteria forming the biofilm.

## 2. Technical Background

In the late 18th century, the direct piezoelectric effect was discovered by Pierre and Paul-Jacques Curie, and it describes the accumulation of electrical charge in certain solids in response to applied mechanical stress. Conversely, materials as crystal quartz respond to an applied electrical field proportionally with mechanical deformation. This phenomenon, known as the “inverse piezoelectric effect,” was discovered by the physicist Gabriel Lippmann in 1881. However, the first practical application of piezoelectric crystals in ultrasonic transducers was implemented by Langevin in 1915 and it was only in the 1950s that crystal quartz was substituted with piezoceramics opening the gate for the development of commercial ultrasonic applications [22].

Ultrasonic processes are characterized by the generation of sound waves at frequencies above the audible range for human beings. The use of ultrasound waves is diverse and ranges from the industrial technology, such as precision processing or semiconductor production, over or underwater communication, to the field of medicine where it is used, e.g., for diagnostic and sterilization procedures. The most common application represents the ultrasonic cleaner which generates and transfers ultrasound waves, usually ranging from 20–40 kHz to a fluid. The agitation of the fluid leads to the formation of quickly collapsing gas bubbles within the fluid that ablate adherent contamination [23]. The existing ultrasonic toothbrushes use working frequencies from 20 kHz up to 10 MHz, depending on the manufacturer.

One of the main physical processes involved in ultrasonic cleaning is the cavitation effect, which includes initiation, growth, oscillation, and collapse of gas bubbles, resulting in significant mechanical forces responsible for permanent chemical and physical changes on the surfaces. The principle of cavitation can be categorized into stable and inertial cavitation. Stable cavitation means the use of low-intensity ultrasound energy to induce stable, resonant oscillations of the already existing microbubbles, so that shear forces act within the fluid flow. By increasing the ultrasound intensity, the microbubbles start to grow and finally collapse, giving a lot of heat to the inrushing fluid—the process referred to as inertial cavitation.

The core technology of ultrasonic toothbrushes did not fundamentally alter since its first publication in 1992 by the inventor Robert T. Bock [24]. The main idea is based on the inverse piezoelectric effect where a piezoelectric crystal resonates and mechanically deforms in the mouth cavity due to the applied pulsed electrical field. Conversion from electrical to mechanical energy results in the propagation of ultrasonic waves that enforce the movement of the bristle tips.

Bock described three possible approaches for the removal of dental plaque from the teeth surface. The first approach involves placing an ultrasonic transducer directly in the mouth and allowing the waves to propagate through the fluid. It is comparable to the principle of the ultrasonic cleaner, where the transducer is supposed to be covered with the fluid completely. The movement of the transducer is not controllable and can potentially cause oral damage. The second approach, which is used widely in the current ultrasonic toothbrush technology, involves the use of ultrasonic energy to excite vibrations in the head of the toothbrush. The last approach describes a high energy device that is only used by professional dentists where the vibrations will be transmitted to a metallic tip (so-called ultrasonic scaler).

The first ultrasonic toothbrushes were constructed by placing an ultrasonic transducer into the head of a commercial toothbrush so that the mechanical deformation energy of the piezo-crystal could be transmitted to the bristles. The resulting mild cavitation in the dentifrice was supposed to agitate dental plaque on the surface of teeth and gingiva.

The propagation speed of ultrasound is dependent on the medium, i.e., in a gaseous medium, the ability to propagate is low. Liquids allow higher propagation speeds and solids display the highest ones. Dental fluid during tooth brushing is usually a mixture of liquids (water, saliva, and liquidated toothpaste) with entrapped air bubbles. The ultrasound wave is propagating through several liquid-air interfaces, consequently leading to a significant reduction of the intensity compared to the conditions in an ultrasonic cleaner.

Although earlier attempts primarily were aimed at distributing the ultrasonic waves energy through the head of the toothbrush, the bristles, or the bubbly fluid between the bristles, this approach sometimes resulted in a reduction of energy propagation rather than facilitating bubble formation. Brewer [25] published a method where sound waves are not directly transmitted through the bristles but to an acoustic waveguide. This acoustic waveguide focuses the waves and pushes them forward to increase the dental fluid flow to the intensities and velocities needed for efficient microbubble production. The combination of the transducer and waveguide enables directed formation of microbubbles and well-controlled acoustic streaming.

**Figure 2:**
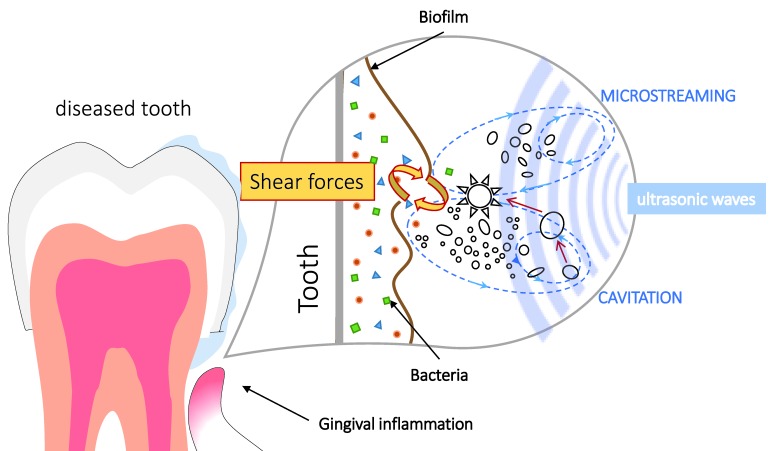
Factors influencing dental biofilm dynamics.

Comprehension of the mechanisms underlying the dental biofilm removal by ultrasonic waves presupposes a better understanding of the biological and physical interactions involved (Figure 2). The main factors for biofilm formation are chemical, physicochemical, and hydrodynamic properties of the medium, the degree of surface preconditioning by organic films, and the capability of the microorganism to perform adhesion [26]. Now we can consider some bio-physical effects that act on these factors and influence the microbial formation, as well as the ultrasound-assisted biofilm detachment.

## 3. Hydrodynamic Effects Involved in Toothbrushing

Manual toothbrushes can be effective in teeth cleaning. This oldest toothbrushing method depends on the type of toothbrush, personal motivation, brushing motions, and manual agility. Saruttichart et al. compared performances of a manual toothbrush and an ultrasonic toothbrush (without bristle motion) in patients with fixed orthodontic appliances in terms of reducing plaque and microbial load (in particular *S. mutans*) and gingival inflammation [27]. Over 30 days, the manual toothbrush performed significantly better than the motionless ultrasonic toothbrush in removing plaque on the bracket side, whereas no difference in the gingival status or numbers of streptococci could be observed.

On the other hand, many other available studies report on significantly better removal of plaque by oscillating and/or rotating ultrasound toothbrushes compared to manual ones. In the short-term study by Costa [28], the action of an ultrasonic, an oscillatory-rotary, and a manual toothbrush was investigated. Patients with orthodontic appliances (*n* = 21) were divided into three groups according to the toothbrushing. The ultrasonic and sonic brush removed noticeably more *S. mutans* than the manual one. A similar conclusion was made by Zimmer et al. [29] based on their observations over a period of 30 days for a larger participant group (*n* = 64).

It can be concluded that correctly performed manual toothbrushing is effective but can be outperformed by ultrasonic cleaning, provided the acoustic action is combined with mechanical movements.

Powered toothbrushes are beneficial for patients lacking manual dexterity or for simplifying self-performed oral hygiene [27]. Regarding their mode of action, powered toothbrushes can be grouped as shown in Table 1. That is, the side-to-side action mode where the brush head moves laterally, counter-oscillation mode where each group of bristles rotates in opposite directions, rotational oscillation mode where the whole brush head rotates in one direction and then in the other, circular action mode where the head rotates in one direction, ultrasonic mode where the bristles are vibrating at ultrasonic frequencies, and ionic mode where an electrical charge is applied to the tooth surface [20].

Independent on the action mode, cavitational effects seem to always be present upon brushing to a greater or lesser extent. The cavitational removal of bacteria from a dental surface occurs in a three-phase system of air bubbles, fluid, and solid surface. The desired ultrasonic cavitation occurs at higher intensity levels and lower frequencies [26], and it is additionally affected by the temperature, medium concentration, pHm and exposure time [23]. The cavitation itself is accompanied by local shock waves, temperature gradients, and free radical generation. Moreover, the resulting laminar flow causes high liquid shear forces that may lead to the destruction of bacteria and their extracellular matrix, even in gingival regions.

The biofilm strength (both cohesive and adhesive) depends on the physical properties of the tooth surface, where irregularities provide more space for bacteria to colonize and to be more protected from hydrodynamic forces. Hydrodynamic forces acting in the oral cavity (wall shear forces) are caused by (a) fluid flow and (b) cavitation effects of entrapped air bubbles in the dental fluid. The turbulent fluid flow of oral fluid usually results from the bristle motion at high frequencies. The application of acoustic energy alone (without bristles) may generate shear forces due to the formation of water streams and shock waves. However, the degree of contribution of ultrasound waves to oral biofilm reduction needs to be studied carefully [13]. The qualitative and quantitative comparisons of manual, sonic, and ultrasonic toothbrushes have been performed in the short-term (Table 2), single-brushing (Table 3), and in-vitro (Table 4) studies.

Bussher et al. [21] reported that acoustic pressure waves generated by powered toothbrushes could transfer energy up to a distance of 6 mm. For this purpose, a 3D oscillating-rotating brush (73 Hz) and a sonic toothbrush (250 Hz) have been evaluated in non-contact removal of oral biofilm. Both brushes generated fluid flow and the inclusion of air bubbles, but they significantly lost non-contact acoustic energy transfer at 2 mm to 4 mm. The authors proposed two possible mechanisms to explain this effect. The first mechanism stated that absorbed acoustic energy led to a viscoelastic expansion of biofilm and the resulting stress destroyed the dental plaque. Another mechanism describes the detachment of the whole biofilm when the deformation is in the plastic range but below the yield point. Thus, either the growing biofilm is absorbing the transferred energy and is only partly removed/destroyed, or the energy only breaks down the adhesive contact between bacteria and substratum. The same authors also conclude that the reduction of oral biofilm is more efficient at small distances between bristles and tooth surfaces, as well as at higher frequencies.

The systematic review of Schmidt [13] showed that the biofilm was not reduced significantly without direct contact of the bristles with the teeth surface. Therefore, the reduction of biofilm without mechanical work by bristles and only by the application of acoustic waves is possible. However, based on the existing literature, it can be assumed that the combination of sonic and ultrasonic action synergistically increases the effective cleaning of teeth [30,31].

Takenouchi et al. showed that ultrasonic toothbrushes were more effective in decreasing dental microbial load and increasing the flow rate of the dental fluid over a study time range of four weeks [32]. The ultrasonically activated water stream may remove dental biofilm even in non-contact surfaces by stimulating saliva flow. Nevertheless, the efficient removal from periodontal pockets, and therefore, the improvement of gingival status, could not be shown in that short-term study. In general, the efficacy of ultrasonic toothbrushes could not be assigned to acoustic waves but rather to the mechanical action by ultrasound-induced vibration of the bristles on the brush head.

The short-term study of Forgas–Brockmann et al. [33] evaluated an ultrasonic toothbrush from Ultrasonex^®^ (Sonex International Corp., Brewster, NY, USA) with Oral-B sonic toothbrushes and showed no significant difference in the reduction of gingival symptoms. Both test groups showed a significant reduction but no complete elimination after 30 days. The long-term study by Lv et al. [34] compared a high-frequency sonic power toothbrush with an oscillating-rotating power toothbrush and a traditional sonic toothbrush in reducing plaque and gingivitis. The evaluation was based on the Rustogi Modified Navy Plaque Index (RMNPI), Modified Gingival Index (MGI), as well as on the Gingival Bleeding Index (GBI). In total, 119 subjects included in the study. The high frequency sonic power toothbrush was not significantly different from the oscillating-rotating power toothbrush in gingivitis reduction while it demonstrated statistically significantly greater reductions in plaque after 6 months.

In the single-brushing study of Anas et al. [17], students at a dental school in Casablanca applied the ultrasonic toothbrush at an angle of 45° to the tooth axis in the gingival direction and observed a better but not statistically significant (*p* = 0.098) result in comparison to oscillatory-rotary toothbrushes. However, all compared modes of actions: manual, oscillatory-rotary, and ultrasound showed an enhancement in dental biofilm decomposition that may correspond to the instructions that the participants received, and accordingly, their oral hygiene may have been conducted better. Costa [28] who compared all three modes of action as well, classified the sonic and ultrasonic as being equally good in reducing gingival inflammation and *S. mutans*. However, the ultrasound treatment showed a better result in the removal of plaque.

In another study, Biesbrock et al. [35] evaluated in a single-brushing study, the effectiveness of a motionless and active ultrasonic toothbrush. The motionless toothbrush removed significantly less plaque than the actively moving toothbrush, which partially correlates with the results of Saruttichart’s group.

In favor of the ultrasound treatment and consistent with the statements of Anas [17] are the results by Horiushi et al. [36], who compared different vibration modes by noncontact brushing in-vitro. For this purpose, streptococci grown on pellets were used as dental biofilm substitute and exposed to four sonic action modes: pulsed ultrasound with sonic vibration, continuous ultrasound with sonic vibration, sonic vibration only and no ultrasound, and no sonic as the control group (manual). After 3 min, the residual biofilm and the amount of water-insoluble glucan were measured. The pulsed ultrasound mode showed more reduction of biofilm (68%) compared to the continuous ultrasound mode (46%) and the sonic vibration mode only (36%). Still, the difference between the ultrasonic and oscillatory-rotary toothbrush was not statistically significant.

Several further studies (listed in Table 4) evinced a similar state of facts. Mourad et al., in their experimental trials, examined the removal of *S. mutans* from different surfaces with a self-made toothbrush that could apply ultrasonic and sonic frequencies. They showed that the simultaneous application of multiple frequencies displayed the best efficiency [30]. In other words, the combination of sonic and ultrasonic action synergistically increased the hydrodynamic forces that acted on the teeth surface. This effect led to more efficient removal compared to the sole use of one action mode, either ultrasonic or sonic.

Rotational oscillation powered and ultrasonic driven toothbrushes produced the most consistent reduction of plaque and gingivitis in the short and long term. However, additional research and studies are necessary before health professionals can provide patients with evidence-based scientific advice on the relative performance of different powered toothbrushes.

Finally, the mouth hygiene method called water flossing (aka “water picking”) is explicitly based on hydrodynamic forces since it applies streams of water sprayed in steady pulses. The water jet, acting like traditional teeth floss, removes plaque and trapped food resting between the teeth [37]. The main advantages of water flossing are easiness to use (especially for people with braces or bridges) and the massage action that positively contributes to gum health. In the randomized two-group pilot study by Lyle et al., water flossing in combination with manual toothbrushing showed significantly higher plaque removal ability in comparison to the interdental brushing/manual toothbrushing group [38].

Water flossers are helpful for people with dexterity issues, such as arthritis, who find string flossing difficult. Among the disadvantages could be the high price and large space required for storage. Jolkovsky and Lyle in their recent review extensively addressed the water flosser safety issues and disproved suggestions of possible detrimental effects of this technique [39].

## 4. Conclusions and Prospects

Our aims were (a) to briefly summarize the existing knowledge on dental biofilm formation and its removal, and to (b) overview the existing experimental and clinical studies on the efficacy of ultrasonic toothbrushes with the estimation of their potential. Included studies were considered based on the technical and microbiological aspects of ultrasound applications in the oral cavity, as well as in terms of quantitative biofilm removal, hydrodynamic phenomena, and the improvement of oral health-related to the reduction of plaque and gingival inflammation.

It is challenging to assess the efficiency of different toothbrushes, as there are so many factors contributing to the removal of plaque and the maintenance of oral hygiene. Nevertheless, published data indicate that ultrasonic, as well as electric toothbrushes, maintain better oral hygiene than manual counterparts. By direct comparison of different toothbrushing systems, most clinical trials show that the sonic/ultrasonic toothbrushes perform better than purely non-acoustic powered brushes. Sonic/ultrasonic toothbrushes may have the potential to reduce more dental biofilm by stimulating more hydrodynamic effects and also by forcing more efficient brushing motions.

The core technology of ultrasonic toothbrushes remains the ultrasound-induced vibration of the bristles. The analysis of the reports and reviews published in recent years allows us to conclude that the mechanical energy transferred from the piezoelectric element leads to the vibration of the bristles. This primarily but not solely causes the cleaning action in the oral cavity. In addition to evoking high-frequency bristle motions, ultrasonic waves induce high velocity flows of oral fluids and additional acoustic microstreaming which indicates a better removal of dental biofilm than the sole application of mechanical scratching or sonic frequencies.

The differences in the reduction of dental plaque by ultrasonic vs. sonic toothbrushes were not statistically significant. Both types of toothbrushes showed successful removal of plaque and reduction in gingival infection but no elimination of already existing periodontal diseases, as well as no difference in the infiltration of supra- and sub-gingival regions. The combination of both types, sonic and ultrasonic, showed the most promising result in maintaining good oral health.

Although the ultrasonic cleaners also use the acoustic energy in a liquid medium, they are not directly comparable to ultrasonic toothbrushes since acoustic waves in the mouth are propagating through a multiphase system. Further investigations are needed to clarify the correlation between the manner of application of acoustic waves on the teeth surface (e.g., sonic intensities, frequency combinations, wave modulations, etc.) and the resulting hydrodynamic forces.

An interesting and promising trend in oral hygiene technologies could be hybrid solutions, combining different physical mechanisms for more efficient plaque removal. Waterpik^®^ Sonic-Fusion-Technology is based on the synergy between sonic toothbrushes and the water flosser. The toothbrush head contains a built-in water flosser tip. To determine the plaque reducing effectiveness of the Sonic-Fusion device, Qaqish et al. carried out an experimental study and found that the Sonic–Fusion^®^ group was more than twice as effective as the standard brushing and flossing group, for all measurements [40].

Future approaches may also include analysis of the ultrasound signals for estimation of cleaning efficacy. Such a technology has been already described by Zhang et al. [41] and detects the signal from reflected ultrasound waves, which is then converted by an ultrasound transceiver to a non-linear peak with respect to time. Therefore, the surfaces of the tooth and the biofilm provide two time-resolved peaks, with the biofilm’s peak arriving earlier. If two peaks are measured, the toothbrush gives feedback to the user via a light-emitting diodes (LED) panel.

Another interesting, current trend is piezoelectric ultrasound tooth-whitening devices that utilize the same hydrodynamic effects but additionally change the chemical properties of the biofilm to whiten the teeth. In comparison to the conventional whitening method which uses LED light, an ultrasound whitening apparatus works in combination with hydrogen peroxide and carbamide peroxide. The ultrasound-driven decomposition of these chemicals as they get exposed to a resonant frequency (1.6–1.8 MHz) in the oral cavity leads to an in-situ release of reactive oxygen species that enter the enamel and dentin and bleach (and also sterilize) the teeth surface. The accompanying pressure and temperature gradients lead to the damage of the bacterial biomass. The ultrasonic whitening procedure distinguishes itself by a significantly shorter operation time and fewer toxic effects [23]. Further investigations in this direction will seemingly focus on the optimization of bleaching chemicals in toothpaste.

## 5. Methodological Remarks

Although this paper represents a thematic rather than a systematic literature review, the authors express their commitment to Cochrane’s fundamental principles and try to keep this study close to the PRISMA protocol guidelines developed for better transparency of systematic reviews. A comprehensive literature search was carried out in Web of Science, PubMed, and Google Scholar databases up to 01-02-2020 (the date when the searches were last performed). The performed queries can be divided into three separate search groups, addressing the three main thematic domains of this review: (1) “oral microbiota,” “dental plaque,” “dental biofilm”; (2) “toothbrushing,” “powered toothbrushes” “dental plaque removal” and more specifically (3) “sonic toothbrushing” and “ultrasonic toothbrushing.” Only articles published in English and related to the study topic were included in this review.

## Figures and Tables

**Table 1:** Different types of powered toothbrushes and their working frequencies.

Type of Toothbrush	Mode of Action	Frequency of Movement	Examples
Lateral motion	The brush head moves forth and back	300 to 600 min^−1^	Oral-B 35
Counter oscillation	Adjacent tufts of bristles (usually six to ten) rotate in one direction and then counter-rotate in the opposite direction	Up to 48,000 min^−1^	Oral-B Ultra Plaque Remover
Rotation oscillation	The whole brush head is rotating in one direction and then the other	Up to 62,000 min^−1^	Oral-B Triumph, Oral-B vitality 2D
Circular	Brush head rotates in only one direction	24,000—48,000 min^−1^	Philips Sonicare
Ionic	An electrical charge is applied to the tooth surface by generating ions in the oral cavity	Up to 31,000 min^−1^	Dr. Tung’s
Water flosser	A targeted stream of water removes plaque, food particles.	1200–1400 min^−1^	Sidekick^®^ (Water Pik, Inc); Oral irrigator (Panasonic Co.)
Ultrasound	The filaments of the brush head vibrating at ultrasound frequencies	mostly 10^8^ min^−1^ (corresponds to 1.6 MHz)	Ultrasonex, Curaprox

**Table 2:** Short-term studies on sonic toothbrushing effectiveness.

Name, Year	Methodology	Participants	Intervention	Result
Forgas–Brockmann, 1998	Examination on day 0, 15 and 30 2 groups Scoring by GI, BI, PI ^1^	*n* = 62 at least 16 healthy teeth no orthodontic appliances PI ≥ 2; BI ≥ 0.5	Ultrasonex Ultrasound 1.6 MHz Oral-B Sonic	Both showed a reduction of gingival inflammation (GI) No significant difference
Zimmer, 2002	Examination on day 0, 30 and 60 2 groups Scoring by PI ^1^, API ^1^	*n* = 64, 32 male, 32 female	Ultra Sonex Ultima Ultrasound vs. Manual	The ultrasonic toothbrush showed significantly better removal of plaque.
Saruttichart, 2017	Examination on day 30, then switch to other toothbrushes for further 30 days. 2 groups Scoring by PI ^1^, GI ^1^, amount of *S. mutans*	*n* = 25 patients with orthodontic appliances	Comparison of modes: Manual Ultrasonic Motionless Ultrasonic	The manual toothbrush performed better, but no difference in *S. mutans* removal
Costa, 2006	Examination on day 15 (own toothbrush), switch to a new toothbrush and examine on day 30 (1/3 toothbrush), 45 (own toothbrush), etc. 3 groups Scoring by PI ^1^, GI ^1^, amount of *S. mutans*	*n* = 21 patients with orthodontic appliances with instructions Group 1: ultrasonic/sonic/manual Group 2: manual/US/sonic Group 3: sonic/manual/US	Ultra Sonex Ultima Ultrasound Oral-B 3D Sonic Oral-B 30 manual	3D and Ultima removed *S. mutans* better than the manual brush. Ultima showed significantly higher PI scores on the bracket side. No difference in reducing GI ^1^ or amount of *S. mutans*
Goyal, 2007	Examination on day 30 2 groups Scoring by oral examination and questionnaires	*n* = 53 *n* = 26: US ^1^ *n* = 27: manual Restrictions: Mild to moderate gingivitis (GI ≥ 1.5) Minimum of 18 natural teeth	Ultreo Ultrasound Oral-B 35-MTB Manual	Oral examination: No significant differences in GI All groups showed a significant reduction in gingival inflammation Ultrasound scored better by GI in comparison to the manual toothbrush

^1^ GI—gingival inflammation; BI—bleeding index; US—ultrasound; PI—plaque index; API—approximal plaque index.

**Table 3:** Single-brushing studies on the sonic toothbrushing effectiveness.

Name, Year	Methodology	Intervention	Result
Biesbrock, 2008	4 groups, *n* = 31 1 Group: 2 min brushing with US, with instructions 2 Group: 2 min brushing without ultrasound 3 Group: 2 min holding by a professional dentist at a 3 mm distance 4 Group (Control) Rubbing of toothpaste without a toothbrush Scoring by API ^1^	Ultrasound Compared modes: Motionless and active	US showed significantly better performance in plaque removal compared to the control group (*p* < 0.001). Group 4 and 3 showed no difference. The first group compared to the second group had a 12.4% higher plaque removal score (*p* < 0.001)
Anas, 2018	*n* = 50 students at a dental school in good general health 12 h no oral hygiene before start	Curaprox CHS Mode: soft With 32,000 to 42,000 oscillations/min Oral-B vitality 2D Rotational-oscillatory Colgate Extra clean manual	All brushes showed a reduction of the plaque index US and sonic performed significantly better than the manual brush Difference between US and sonic is not significant

^1^ US—ultrasound; API—approximal plaque index.

**Table 4:** In-vitro studies on the sonic toothbrushing effectiveness.

Name, Year	Methodology	Intervention	Result
Mourad, 2007	Examination of *Streptococcus mutans* adherent to various surfaces	Self-prepared toothbrush: Ultrasound and sonic processes can be individually modified and applied	The combination of both showed the successful removal of *S. mutans*
Sorensen, 2008	Examination of the tooth surface and restoration integrity using scanning electron microscopy *n* = 60 of human molars *n* = 33: orthodontic *n* = 32: crown	Ultreo Ultrasound Oral-B Triumph Oscillating-rotating Oral-B 35 Manual Unbrushed (control)	No safety concerns with any treatment-related to orthodontic or crown appliances were identified
Horiuchi, 2018	Examination after 3 min non-contact brushing. Measurement of water-insoluble glucan and residual biofilm observed by scanning electron microscopy	Compared modes: 1 pulsed ultrasound with sonic vibration 2 continuous ultrasound waves with sonic vibration 3 sonic vibration only 4 no ultrasound nor sonic vibration (control)	The most reduction showed mode 1. Sonic and ultrasonic treatment was significantly better than the manual. Ultrasound showed no significantly better removal than the oscillatory-rotary mode.
Robert, 2010	Single-brushing study Examination of biofilm adherent on apatite disks using digital image analysis Without contact of bristles A distance of 3 mm	Compared modes: 1 sonic and ultrasonic vibration 2 only sonic vibration of the ultrasonic toothbrush 3 normal sonic vibration 4 oscillatory-rotary action 5 held in toothpaste	All modes exhibited some removal of biofilm The combined mode 1 with the ultrasound showed the greatest reduction.
